# Prognostic Role of *TP53* and *ARID1A* Mutations in Alcohol-Consuming Patients With Uterine Endometrioid Carcinoma

**DOI:** 10.1097/PAI.0000000000001343

**Published:** 2026-07-13

**Authors:** Yasemin Adalı

**Affiliations:** * Centre for Public Health, Queen’s University Belfast, School of Medicine, Dentistry and Biomedical Sciences, Belfast, United Kingdom; † Department of Epidemiology, Centre for Public Health, Pamukkale University Faculty of Medicine, Denizli, Türkiye

**Keywords:** uterine endometrioid carcinoma, alcohol consumption, TP53, ARID1A, TCGA, survival, epidemiology

## Abstract

Uterine endometrioid carcinoma (UEC) is the most common subtype of endometrial cancer. This study aimed to investigate the prognostic relevance of *TP53* and *ARID1A* mutations in alcohol-consuming UEC patients using publicly available TCGA data. A total of 207 UEC patients were identified from the TCGA CPTAC-3 project. Alcohol consumption status was extracted from self-reported clinical data. Patients were grouped into alcohol consumers and nonconsumers. Kaplan-Meier survival estimates and multivariable Cox proportional hazards models were used to evaluate the association between gene mutation status and overall survival, adjusted for age, stage, and grade. Sensitivity analyses excluded deaths within the first year. Out of 207 patients, 47 (22.7%) reported alcohol consumption. *TP53* mutations were more prevalent in alcohol consumers compared with nonconsumers (36.2% vs. 21.3%, *P*=0.037). In the alcohol-consuming subgroup, patients with wild-type *TP53* had significantly worse survival than those with *TP53* mutations in sensitivity analysis (HR=2.85; 95% CI: 1.01-8.05; *P*=0.047). *ARID1A* wild-type status was associated with increased mortality risk compared with mutated *ARID1A* in adjusted models (HR=4.00; 95% CI: 1.45-11.01; *P*=0.007). No statistically significant survival differences were found for *PTEN*, *PIK3CA*, or *KRAS* mutations. *TP53* and *ARID1A* mutation status may have prognostic relevance among alcohol-consuming patients with UEC. These findings suggest a possible gene-environment interaction in endometrial cancer and support the need for further validation through prospective studies.

Uterine endometrioid carcinoma (UEC) represents the most common histologic subtype of endometrial cancer, accounting for ∼75 to 80% of cases and predominantly affecting postmenopausal women.^1^ Although UEC is generally associated with favorable clinical outcomes, substantial heterogeneity in prognosis exists, reflecting differences in tumor biology, stage at diagnosis, and patient-related factors. While established clinicopathological features such as tumor grade and stage remain central to risk stratification, growing evidence highlights the importance of molecular alterations and lifestyle-related exposures in modulating disease progression and survival outcomes.^2–4^

Alcohol consumption is a known risk factor for several cancers, including breast, liver, and colorectal malignancies.^5^ Its role in endometrial carcinogenesis, however, remains less well defined. Alcohol may influence cancer development and progression through various mechanisms such as increasing estrogen levels, generating reactive oxygen species, and impairing DNA repair.^6,7^ Several studies have evaluated the association between alcohol intake and endometrial cancer risk, but its impact on tumor molecular characteristics and patient survival remains underexplored.^7^

Recent genomic analyses have identified frequent mutations in genes such as *PTEN*, *ARID1A*, *PIK3CA*, *TP53*, and *KRAS* in endometrioid endometrial carcinoma, each with potential prognostic or therapeutic relevance.^8,9^ Among these, *TP53* mutations are often associated with more aggressive disease and poor survival, while *PTEN* and *PIK3CA* alterations are common in early-stage tumors with better outcomes.^10,11^ Nonetheless, limited evidence exists regarding the interaction between alcohol consumption and such mutations in determining clinical outcomes.

The Cancer Genome Atlas (TCGA) project has enabled comprehensive genomic profiling of uterine cancers and provides an opportunity to assess the impact of lifestyle factors in a molecular context. In this study, we aimed to investigate the relationship between alcohol consumption and overall survival in uterine endometrioid carcinoma patients, stratified by key molecular biomarkers using publicly available TCGA data. We hypothesized that alcohol intake may modulate the prognostic value of gene mutations such as *TP53* and *ARID1A* in this population.

## PATIENTS AND METHODS

### Data Sources and Patient Selection

Launched by the National Cancer Institute and the National Human Genome Research Institute, the TCGA project is a landmark initiative designed to comprehensively elucidate the molecular changes occurring during carcinogenesis and tumor progression, while linking cancer genomic data to patients’ clinical information, including survival status. For the study, the clinical data and alcohol usage status of patients from the TCGA CPTAC-3 project were obtained from the Genomics Data Commons (GDC) data repository (https://portal.gdc.cancer.gov/;^12^ accessed on 15 January 2025). This cohort included 207 patients based on their documented alcohol consumption status. Since the data were extracted from a publicly available database, no ethics committee approval was required. This study was a retrospective analysis using data from a public resource.

### Term of Definition

Data on uterine endometrioid carcinoma patients were obtained from the Cancer Genome Atlas (TCGA) program and selected based on alcohol consumption status. A total of 207 patients had recorded alcohol consumption data and were categorized as having a history of alcohol consumption or no history of alcohol consumption. The staging of all patients was determined according to the American Joint Committee on Cancer (AJCC) Clinical Group Staging System, based on the version used at the time of diagnosis, and classified as early stage (stage I to II) or advanced stage (stage III to IV). TNM staging was also assigned according to the version applicable at the time of diagnosis. In the original data set, tumor grading was classified as G1, G2, and G3; however, for the analysis, it was regrouped into G1 and G2 as low grade and G3 as high grade. In addition, postoperative adjuvant treatments were examined, revealing that the TCGA data set recorded supplementary treatment approaches such as radiotherapy, immunotherapy, and radiation therapies. Furthermore, biomarker data for *TP53*, *PTEN*, *ARID1A*, *PIK3CA*, and *KRAS* were analyzed and classified as mutated or wild type.

### Follow-Up and Outcome Measures

In the original TCGA data set, follow-up duration was recorded in days. For the eligible patient cohort, the mean follow-up time corresponded to ∼3.23 years. The primary outcome of interest was overall survival (OS), defined as the time from the initial pathologic diagnosis to death from any cause. Patients who were alive at the last available follow-up were censored at that time in the survival analyses.

### Statistical Analysis

Categorical variables were compared using the χ² test or the Fisher exact test, whereas the Student *t* test or the Mann-Whitney *U* test was applied for continuous variables. Survival analysis was conducted using the Kaplan-Meier method, with differences assessed through the log-rank test. Multivariate analyses were performed using the Cox proportional hazards model to evaluate independent prognostic factors. Hazard ratios (HRs) with 95% CIs were calculated through Cox proportional hazards models, adjusting for age at diagnosis, tumor grade, and disease stage. A *P*-value of <0.05 was considered indicative of statistical significance. In addition, a sensitivity analysis was carried out by excluding patients who died within the first year of follow-up to reduce the potential influence of reverse causation on the observed associations.

### Differential Expression and Functional Enrichment Analysis

To investigate the biological context associated with *TP53* and *ARID1A* mutation status, transcriptomic analyses were performed using RNA sequencing data from TCGA-UCEC. Gene expression quantification (STAR-counts) was retrieved through the TCGAbiolinks package,^13^ and analyses were restricted to primary endometrioid endometrial carcinoma samples with available clinical annotation. Raw count matrices were filtered and normalized using the DESeq2 framework. ^14^ Differential expression analyses were conducted separately for TP53-mutant versus *TP53* wild-type tumors and for *ARID1A* mutant versus *ARID1A* wild-type tumors. Genes with an adjusted *P*-value <0.05 and an absolute log2 fold change ≥1 were considered significantly differentially expressed. Functional pathway enrichment analysis was performed using the enrichR package,^15^ querying the KEGG 2021 Human database.^16^ Enriched pathways were ranked based on adjusted *P*-values and combined enrichment scores, and the top pathways were visualized using dot plot representations. All statistical analyses were conducted using R software (version 4.5.2).

## RESULTS

### Clinicopathological Characteristics

The clinicopathological characteristics of uterine endometrioid carcinoma patients’ data from the TCGA program, stratified by alcohol consumption, are presented in Table 1. A total of 207 uterine endometrioid carcinoma patients from the TCGA data set with documented alcohol consumption status were included in this analysis. Among them, 47 patients (22.7%) reported a history of alcohol consumption, while 160 patients (77.3%) had no history of alcohol intake. The median age at diagnosis did not significantly differ between the 2 groups (*P*=0.473). Specifically, 36.2% of alcohol consumers were diagnosed at ≤60 years, compared with 30.6% of nonconsumers. In addition, a significant difference was observed in race distribution (*P*=0.001), with a higher proportion of White patients (93.6%) in the alcohol consumption group compared with 67.5% in the nonconsumer group. Regarding tumor staging, no statistically significant differences were found between the groups in T stage (*P*=0.290), N stage (*P*=0.254), or M stage (*P*=0.646). Similarly, the distribution of AJCC clinical staging showed no significant association with alcohol consumption, with 78.7% of alcohol consumers and 80.6% of nonconsumers classified as having early-stage disease (*P*=0.774). Furthermore, tumor grade classification did not significantly differ between the groups (*P*=0.333). The proportion of low-grade (grade 1 to 2) tumors was slightly higher in alcohol consumers (80.4% vs. 72.4%), whereas high-grade (grade 3) tumors were more frequent in nonconsumers (26.6% vs. 19.6%). Analysis of treatment modalities revealed that chemotherapy was more frequently administered to alcohol consumers (44.4%) compared with nonconsumers (28.1%), although this difference did not reach statistical significance (*P*=0.225). The primary therapy response also varied between groups but was not statistically significant (*P*=0.201). A complete response to treatment was observed in 77% of alcohol consumers and 89.3% of nonconsumers. In addition, the proportion of patients with persistent disease was slightly higher in the alcohol consumption group (11.5% vs. 6.8%).

**TABLE 1:** Clinicopathological Characteristics of Uterine Endometrioid Carcinoma Patients’ Data From the TCGA Program By Alcohol Consumption

	Alcohol consumption		
Characteristic	No	Yes	*P*
Age at diagnosis, n (%)			
≤60 y	49 (30.6)	17 (36.2)	
>60 y	111 (69.4)	30 (63.8)	0.473
Race, n (%)			
White	108 (67.5)	44 (93.6)	
Non-White	52 (32.5)	3 (6.4)	0.001
T stage, n (%)			
T1	126 (78.7)	34 (72.3)	
T2	14 (8.7)	8 (17)	
T3	19 (11.9)	4 (8.5)	
T4	1 (0.7)	1 (2.2)	0.290
N stage, n (%)			
NX	50 (31.4)	12 (25.5)	
N0	92 (57.9)	26 (55.3)	
N1	13 (8.2)	5 (10.7)	
N2	4 (2.5)	4 (8.5)	0.254
M stage, n (%)			
MX	85 (53.1)	22 (47.8)	
M0	69 (43.1)	21 (45.7)	
M1	6 (3.8)	3 (6.5)	0.646
AJCC stage, n (%)			
Early (stage I-II)	129 (80.6)	37 (78.7)	
Advanced (stage III-IV)	31 (19.4)	10 (21.3)	0.774
Grade, n (%)			
Low (grade 1-2)	116 (72.4)	37 (80.4)	
High (grade 3)	42 (26.6)	9 (19.6)	0.333
Treatment type, n (%)			
Chemotherapy	29 (28.1)	12 (44.4)	
Immunotherapy	2 (2)	0	
Radiation therapy	72 (69.9)	15 (55.6)	0.225
Primary therapy outcome, n (%)			
Complete response	92 (89.3)	20 (77)	
Persistent disease	7 (6.8)	3 (11.5)	
Unknown	4 (3.9)	3 (11.5)	0.201

### Mutation Status of Key Biomarkers

The mutation status of uterine endometrioid carcinoma patients’ data from the TCGA program, stratified by alcohol consumption, is presented in Table 2. The analysis of mutation status for key genes associated with uterine endometrioid carcinoma revealed significant differences in *TP53* mutation status between the 2 groups (*P*=0.037). The mutation rate of *TP53* was higher in alcohol consumers (36.2%) compared with nonconsumers (21.3%). No significant differences were observed in the mutation status of *PTEN* (*P*=0.613), *ARID1A* (*P*=0.392), or *KRAS* (*P*=0.337) between the groups. However, a marginally significant difference was noted in *PIK3CA* mutation status (*P*=0.053), with a higher proportion of alcohol consumers having wild-type *PIK3CA* (70.2%) compared with nonconsumers (54.4%).

**TABLE 2:** Mutation Status of Uterine Endometrioid Carcinoma Patients From the TCGA Program According to Alcohol Consumption Status

	Alcohol consumption		
Mutations	No	Yes	*P*
TP53, n (%)			
Wild type	126 (78.7)	30 (63.8)	
Mutated	34 (21.3)	17 (36.2)	0.037
PTEN status, n (%)			
Wild type	32 (20)	11 (23.4)	
Mutated	128 (80)	36 (76.6)	0.613
ARID1A status, n (%)			
Wild type	76 (47.5)	19 (40.4)	
Mutated	84 (52.5)	28 (59.6)	0.392
PIK3CA status, n (%)			
Wild type	87 (54.4)	33 (70.2)	
Mutated	73 (45.6)	14 (29.8)	0.053
KRAS status, n (%)			
Wild type	111 (69.4)	36 (76.6)	
Mutated	49 (30.6)	11 (23.4)	0.337

### Survival Analysis Based on Mutation Status Among Alcohol Consumers

To further evaluate the prognostic significance of key gene mutations in alcohol-consuming uterine endometrioid carcinoma patients, Kaplan-Meier survival analyses were conducted (Fig. 1). The log-rank test was used to compare survival distributions between patients with wild-type and mutated forms of *TP53*, *PTEN*, *ARID1A*, *PIK3CA*, and *KRAS*. A statistically significant difference in overall survival was observed for *TP53* mutation status among alcohol-consuming patients (*P*=0.02), with patients with wild-type *TP53* exhibiting worsesurvival outcomes compared with those with *TP53* mutations. For *PTEN* (*P*=0.34), *ARID1A* (*P*=0.17), *PIK3CA* (*P*=0.85), and *KRAS* (*P*=0.83), no statistically significant differences in overall survival were observed between wild-type and mutated groups.

**FIGURE 1:**
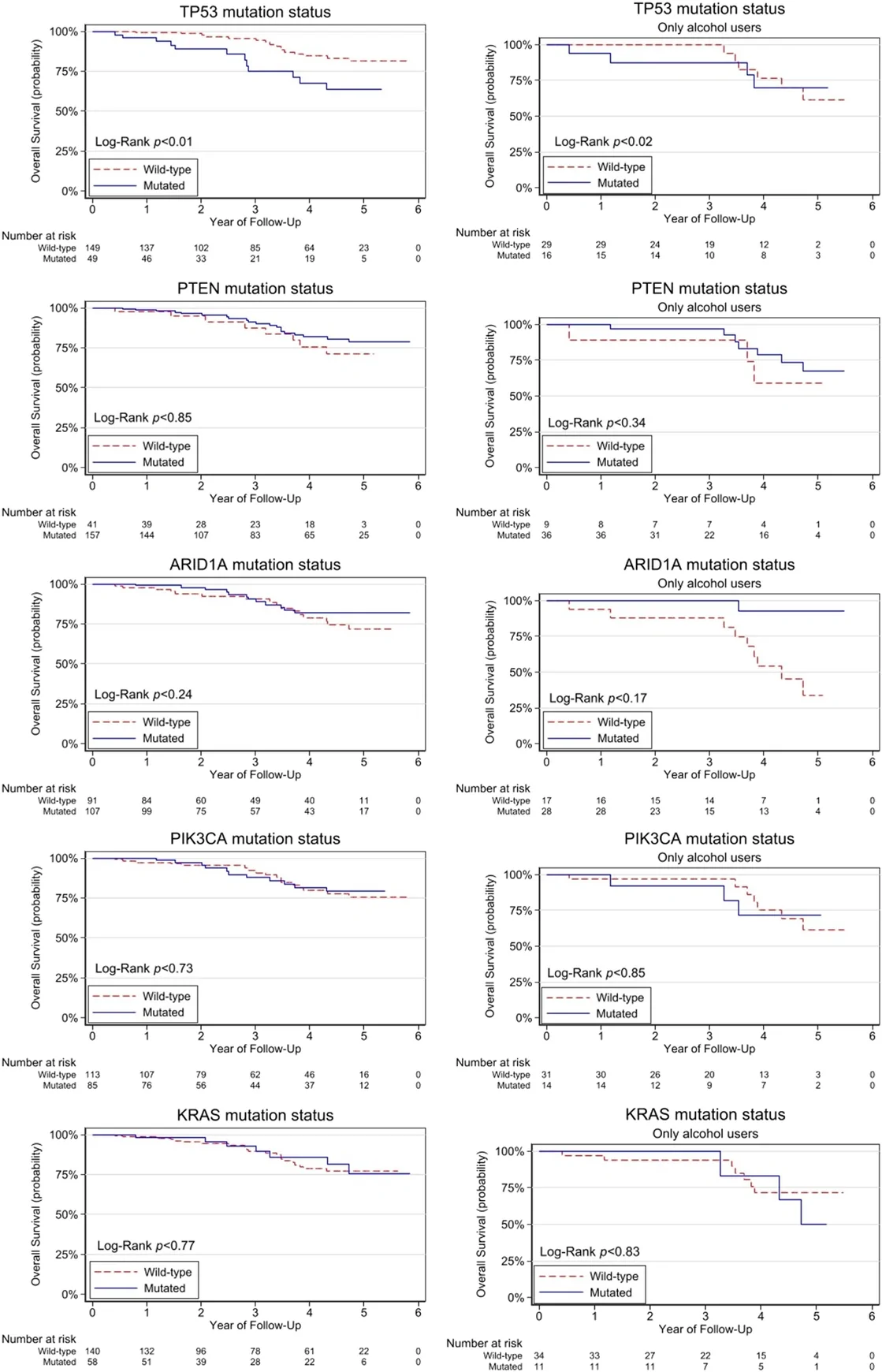
Kaplan–Meier survival analysis according to mutation status and alcohol consumption in uterine endometrioid carcinoma patients from the TCGA program.

### Association Between Alcohol Consumption and Overall Survival in Uterine Endometrioid Carcinoma Patients

The association between alcohol consumption, mutation status, and overall survival in patients with uterine endometrioid carcinoma was evaluated using unadjusted, adjusted, and sensitivity analyses (Table 3). In the *TP53* mutation group, patients with wild-type *TP53* demonstrated poorer survival outcomes compared with those with mutated *TP53* in both unadjusted (HR=2.22, 95% CI: 0.80-6.14, *P*=0.121) and adjusted models (HR=2.60, 95% CI: 0.99-7.19, *P*=0.065). The sensitivity analysis, which excluded patients who died within the first year of follow-up, additionally strengthened this association (HR=2.85, 95% CI: 1.01-8.05, *P*=0.047).

**TABLE 3:** Association Between Alcohol Consumption and Overall Survival in Uterine Endometrioid Carcinoma Patients From the TCGA Program, Stratified By Molecular Biomarkers

	Overall survival							
		Unadjusted		Adjusted*		Adjusted sensitivity analysis†		
Mutations	n/event	HR (95% CI)	*P*	HR (95% CI)	*P*	n/event	HR (95% CI)	*P*
TP53 status								
Wild type	30/6	2.22 (0.80-6.14)	0.121	2.60 (0.99-7.19)	0.065	30/1	2.85 (1.01-8.05)	0.047
Mutated	17/4	0.76 (0.22-2.55)	0.665	0.80 (0.23-2.68)	0.719	17/2	0.65 (0.16-2.55)	0.539
*P-*value for interaction			0.142					0.084
PTEN status								
Wild type	11/3	1.82 (0.43-7.70)	0.413	1.80 (0.41-7.81)	0.432	11/3	1.23 (0.23-6.62)	0.802
Mutated	36/7	1.53 (0.61-3.86)	0.358	1.60 (0.63-4.03)	0.313	36/0	1.87 (0.72-4.86)	0.193
*P-*value for interaction			0.861					0.649
ARID1A status								
Wild type	19/9	4.68 (1.73-12.66)	0.002	4.00 (1.45-11.01)	0.007	19/3	4.28 (1.44-12.66)	0.009
Mutated	28/1	0.24 (0.03-1.869)	0.173	0.30 (0.03-2.37)	0.256	28/0	0.33 (0.04-2.64)	0.301
*P-*value for interaction			0.002					0.004
PIK3CA status								
Wild type	33/7	1.62 (0.61-4.26)	0.327	1.63 (0.61-4.33)	0.325	33/3	1.71 (0.58-4.99)	0.327
Mutated	14/3	1.51 (0.40-5.71)	0.539	2.20 (0.53-8.98)	0.271	14/0	2.20 (0.53-8.98)	0.271
*P-*value for interaction			0.969					0.916
KRAS status								
Wild type	36/7	1.35 (0.54-3.36)	0.509	1.58 (0.63-3.97)	0.322	36/3	1.40 (0.52-3.71)	0.499
Mutated	11/3	2.30 (0.51-10.33)	0.275	1.90 (0.35-10.22)	0.452	11/3	2.22 (0.37-13.22)	0.379
*P-*value for interaction			0.500					0.314

* Multivariable model adjusted for age at diagnosis, grade, and stage status.

† Sensitivity analysis was performed by removing individuals who died within the first year of follow-up.

For ARID1A status, patients with wild-type *ARID1A* showed significantly worse survival outcomes compared with those with mutated *ARID1A* (unadjusted HR=4.68, 95% CI: 1.73-12.66, *P*=0.002; adjusted HR=4.00, 95% CI: 1.45-11.01, *P*=0.007). This association also remained statistically significant in the sensitivity analysis (HR=4.28, 95% CI: 1.44-12.66, *P*=0.009). In addition, no significant associations with overall survival were observed for mutations in *PTEN*, *PIK3CA*, or *KRAS* in either the unadjusted or adjusted models (*P*>0.05). Furthermore, no significant interactions were identified for TP53, PTEN, PIK3CA, or KRAS, whereas a significant interaction was observed for ARID1A status.

### Functional Enrichment Analysis (KEGG)

To explore the biological processes associated with *TP53* and *ARID1A* mutation status, functional pathway enrichment analysis was performed using differentially expressed genes identified between mutant and wild-type tumors. In *TP53* mutant tumors, KEGG pathway enrichment analysis revealed significant enrichment of several biologically relevant pathways, including the estrogen signaling pathway, neuroactive ligand receptor interaction, complement and coagulation cascades, IL 17 signaling pathway, and calcium signaling pathway (Fig. 2). In addition, multiple immune and inflammation related pathways, such as viral protein interaction with cytokine and cytokine receptor and complement activation, were overrepresented. Collectively, these findings indicate that *TP53* mutation status is associated with dysregulation of signaling, immune, and tumor microenvironment-related biological processes.

**FIGURE 2:**
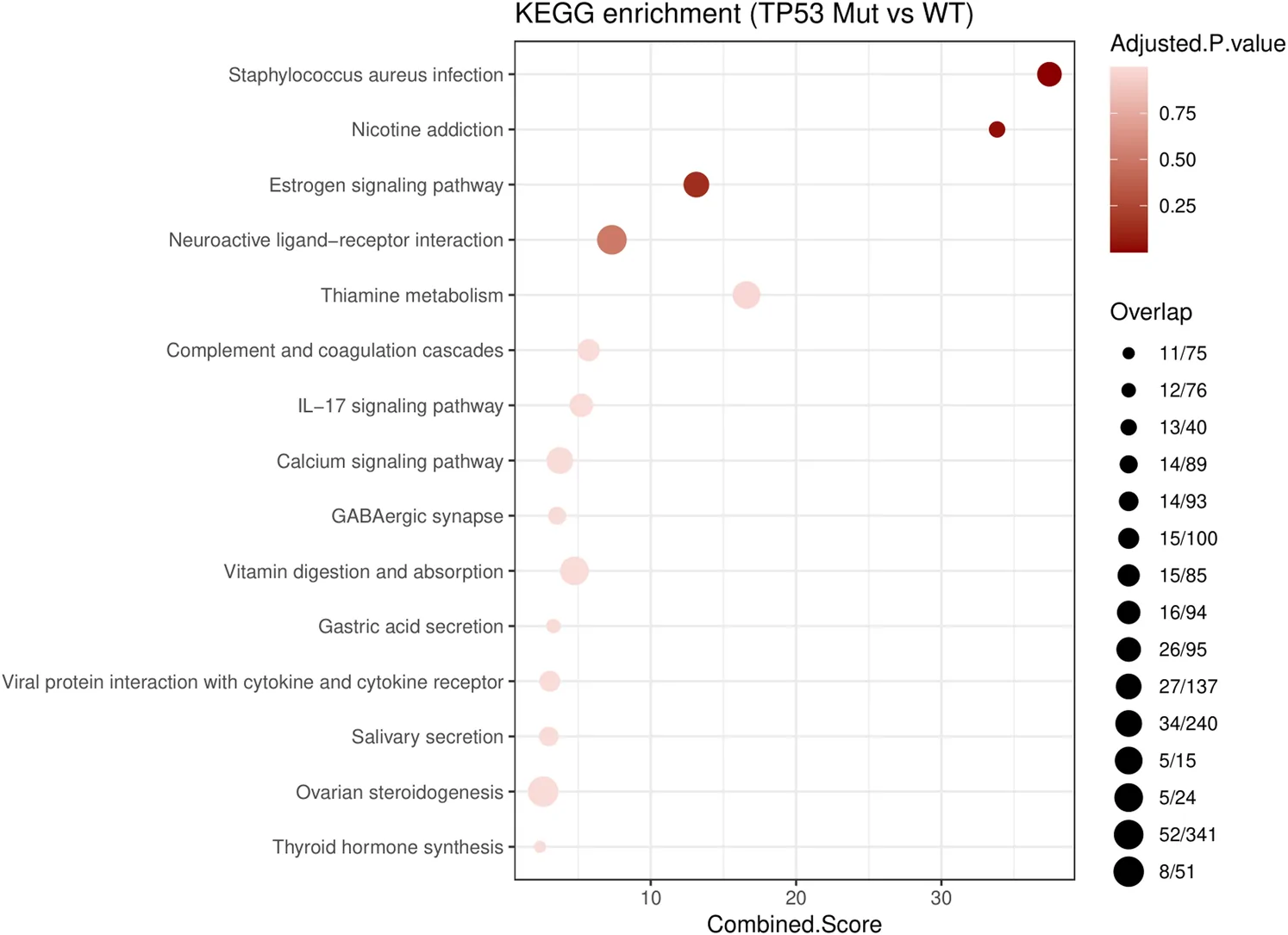
Differential gene expression associated with *TP53* mutation status in uterine endometrioid carcinoma.

In parallel, enrichment analysis based on *ARID1A* mutation status demonstrated predominant involvement of metabolic and differentiation-related pathways (Fig. 3). The most significantly enriched pathways included retinol metabolism, metabolism of xenobiotics by cytochrome P450, pentose and glucuronate interconversions, steroid hormone biosynthesis, lipid metabolism-related pathways, and drug metabolism. These results suggest that *ARID1A* mutations are associated with transcriptional alterations affecting metabolic reprogramming and cellular differentiation-related biological functions. Collectively, these analyses demonstrate that *TP53* and *ARID1A* mutations are associated with distinct yet biologically coherent molecular programs in uterine endometrioid carcinoma.

**FIGURE 3:**
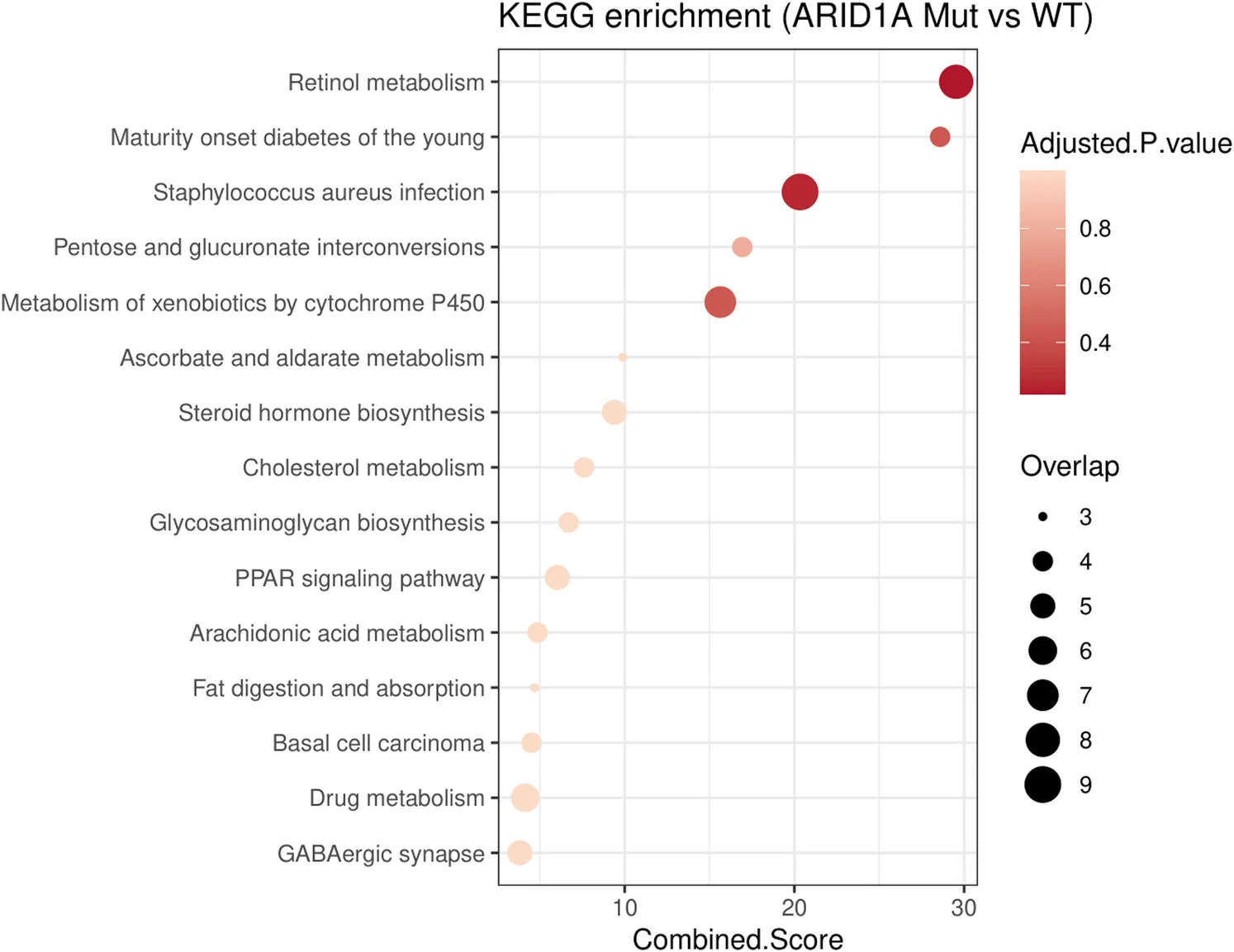
Differential gene expression associated with *ARID1A* mutation status in uterine endometrioid carcinoma.

## DISCUSSION

In this study, we investigated the prognostic relevance of key somatic mutations in the context of alcohol consumption among patients with uterine endometrioid carcinoma using data from the TCGA project. Our findings suggest that *TP53* and *ARID1A* mutations exhibit potential prognostic significance in alcohol-consuming patients, while *PTEN*, *PIK3CA*, and *KRAS* mutations were not significantly associated with overall survival.

The observed association between *TP53* mutation and worse survival outcomes in alcohol consumers aligns with previous literature identifying *TP53* as a key driver of aggressive tumor behavior and poor prognosis across a variety of malignancies, including endometrial cancer.^9,17^ *TP53* mutations are typically enriched in nonendometrioid or high-grade endometrial tumors and are associated with genomic instability and resistance to standard therapies.^18^ Alcohol exposure has been shown to exacerbate genomic instability through the production of acetaldehyde, a toxic metabolite that induces DNA adducts and double-strand breaks.^19^ This may further contribute to tumor progression in *TP53*-mutated cases.

Interestingly, our analysis identified a significant association between *ARID1A* mutation and improved overall survival among alcohol consumers. *ARID1A*, a member of the SWI/SNF chromatin remodeling complex, is commonly mutated in endometrioid endometrial carcinoma and has been implicated in tumor initiation and immune microenvironment modulation.^20^ While *ARID1A* mutations have often been associated with poor prognosis in other cancers, emerging evidence suggests context-dependent effects. For example, in endometrial cancer, some studies have shown that *ARID1A* loss may enhance response to immune checkpoint inhibitors or modulate tumor suppressive pathways, depending on coexisting mutations and environmental exposures.^21,22^ Alcohol’s immunomodulatory effects may interact with *ARID1A-*driven mechanisms, potentially altering the tumor microenvironment and contributing to the observed survival benefit in this subgroup. However, this hypothesis requires further biological validation.

In contrast, *PTEN*, *PIK3CA*, and *KRAS* mutations did not show a statistically significant impact on survival in alcohol-consuming patients. These mutations are frequently observed in early-stage endometrioid tumors and are typically associated with indolent disease.^11^ The lack of association in our analysis may reflect the relatively favorable prognosis of these mutations regardless of alcohol exposure. Alternatively, alcohol’s influence may not be sufficient to modify the tumor biology driven by these alterations.

In addition to the clinical survival associations, our exploratory transcriptomic analyses provide further biological context for the observed findings. Functional enrichment analyses revealed that *TP53* mutant tumors were characterized by enrichment of pathways related to immune and inflammatory signaling, including IL-17 signaling, complement and coagulation cascades, cytokine receptor interactions, as well as hormone-related signaling such as estrogen signaling and broader cellular communication pathways. These observations are consistent with existing evidence indicating that *TP53* mutations are not only drivers of genomic instability but also play a central role in regulating immune responses and tumor microenvironment interactions. Alcohol exposure has been shown to induce oxidative stress, DNA damage, and inflammatory responses that activate *TP53* dependent stress signaling, providing a biologically plausible basis for interactions between alcohol related inflammatory effects and *TP53* associated tumor behavior, which may ultimately influence clinical outcomes.^6,23,24^

Nevertheless, *ARID1A* mutant tumors demonstrated enrichment of pathways primarily related to metabolic and differentiation processes, including retinol metabolism, xenobiotic metabolism by cytochrome P450, lipid-related pathways, and steroid hormone biosynthesis. Dysregulation of these pathways is biologically relevant in hormonally responsive tissues such as the endometrium and suggests that *ARID1A* alterations may influence tumor behavior through transcriptional and metabolic reprogramming. Although direct mechanistic evidence linking alcohol exposure to *ARID1A* alterations remains limited, chronic alcohol consumption has been shown to disrupt retinoid metabolism, detoxification pathways, and lipid homeostasis. These biological processes overlap with pathways enriched in *ARID1A* mutant tumors, thereby providing indirect biological plausibility for a potential interaction between alcohol exposure and *ARID1A-*related tumor biology.^24,25^

Several limitations should be considered when interpreting these findings. First, the retrospective study design and reliance on publicly available, self-reported alcohol consumption data introduce the potential for exposure misclassification and residual confounding. Alcohol use variables in the TCGA database lack information on dose, duration, and frequency, precluding assessment of dose-response relationships that are epidemiologically relevant to cancer progression and survival. In addition, other important lifestyle and clinical factors, including smoking status, body mass index, comorbid conditions, and medication use, were not consistently available and could not be fully adjusted for in the analyses. The generalizability of the findings is also limited, as the TCGA cohort predominantly comprises patients from the United States and may not reflect exposure patterns, genetic backgrounds, or health care contexts in other populations. Finally, given the observational nature of the study, causal inference is not possible. Rather, the results should be interpreted as hypothesis generating, providing a basis for future prospective studies with detailed exposure assessment, improved control of confounding, and integration of molecular and epidemiological data.

## CONCLUSION

In conclusion, our findings suggest that *TP53* and *ARID1A* mutations may be associated with differential survival outcomes among alcohol consuming patients with uterine endometrioid carcinoma, supporting a potential role for gene-environment interactions in shaping disease prognosis at the population level. In addition, exploratory transcriptomic analyses indicate that *TP53* and *ARID1A* mutations are linked to distinct biological pathways related to immune regulation and metabolic reprogramming, providing biological support for the observed epidemiological associations. Future studies with prospective designs, more precise assessment of alcohol exposure and other lifestyle factors, and integrated functional analyses are needed to better elucidate underlying mechanisms, address potential residual confounding, and validate these findings across diverse populations.

## References

[1] Siegel RL Miller KD Wagle NS . Cancer statistics, 2023. CA Cancer J Clin. 2023;73:17–48. 10.3322/caac.21763 36633525

[2] Concin N Matias-Guiu X Vergote I . ESGO/ESTRO/ESP guidelines for the management of patients with endometrial carcinoma. Int J Gynecol Cancer. 2021;31:12–39. 10.1136/ijgc-2020-002230 33397713

[3] Léon-Castillo A . Update in the molecular classification of endometrial carcinoma. Int J Gynecol Cancer. 2023;33:333–342. 10.1136/ijgc-2022-003772 36878561

[4] Crosbie EJ Kitson SJ McAlpine JN . Endometrial cancer. Lancet. 2022;399:1412–1428. 10.1016/S0140-6736(22)00323-3 35397864

[5] Bagnardi V Rota M Botteri E . Alcohol consumption and site-specific cancer risk: a comprehensive dose-response meta-analysis. Br J Cancer. 2015;112:580–593. 10.1038/bjc.2014.579 PMC4453639 25422909

[6] Seitz HK Stickel F . Molecular mechanisms of alcohol-mediated carcinogenesis. Nat Rev Cancer. 2007;7:599–612. 10.1038/nrc2191 17646865

[7] Friberg E Orsini N Mantzoros CS . Alcohol intake and endometrial cancer risk: a meta-analysis of prospective studies. Br J Cancer. 2010;103:127–131. 10.1038/sj.bjc.6605698 PMC2905297 20485288

[8] Murali R Soslow RA Weigelt B . Classification of endometrial carcinoma: more than two types. Lancet Oncol. 2014;15:e268–e278. 10.1016/S1470-2045(13)70591-6 24872110

[9] Getz G Gabriel SB Cibulskis K . Integrated genomic characterization of endometrial carcinoma. Nature. 2013;497:67–73. 10.1038/nature12113 PMC3704730 23636398

[10] Talhouk A McAlpine JN . New classification of endometrial cancers: the development and potential applications of genomic-based classification in research and clinical care. Gynecol Oncol Res Pract. 2016;3:1–12. 10.1186/s40661-016-0035-4 PMC5154099 27999680

[11] Le Gallo M O’Hara AJ Rudd ML . Exome sequencing of serous endometrial tumors identifies recurrent somatic mutations in chromatin-remodeling and ubiquitin ligase complex genes. Nat Genet. 2012;44:1310–1315. 10.1038/ng.2455 PMC3515204 23104009

[12] GDC Data Portal Homepage.

[13] Colaprico A Silva TC Olsen C . TCGAbiolinks: an R/Bioconductor package for integrative analysis of TCGA data. Nucleic Acids Res. 2016;44:e71. 10.1093/nar/gkv1507 PMC4856967 26704973

[14] Love MI Huber W Anders S . Moderated estimation of fold change and dispersion for RNA-seq data with DESeq. Genome Biol. 2014;15:1–21 10.1186/s13059-014-0550-8 PMC4302049 25516281

[15] Kuleshov MV Jones MR Rouillard AD . Enrichr: a comprehensive gene set enrichment analysis web server 2016 update. Nucleic Acids Res. 2016;44(W1):W90–W97. 10.1093/nar/gkw377 PMC4987924 27141961

[16] Kanehisa M Goto S . KEGG: kyoto encyclopedia of genes and genomes. Nucleic Acids Res. 2000;28:27–30. 10.1093/nar/28.1.27 PMC102409 10592173

[17] León-Castillo A Britton H McConechy MK . Interpretation of somatic POLE mutations in endometrial carcinoma. Journal of Pathology. 2020;250:323–335. 10.1002/path.5372 PMC7065171 31829442

[18] Van Gool IC Stelloo E Nout RA . Prognostic significance of L1CAM expression and its association with mutant p53 expression in high-risk endometrial cancer. Mod Pathol. 2016;29:174–181. 10.1038/modpathol.2015.147 26743472

[19] Brooks PJ Zakhari S . Acetaldehyde and the genome: beyond nuclear DNA adducts and carcinogenesis. Environ Mol Mutagen. 2014;55:77–91. 10.1002/em.21824 24282063

[20] Wiegand KC Shah SP Al-Agha OM . ARID1A mutations in endometriosis-associated ovarian carcinomas. N Engl J Med. 2010;363:1532–1543. 10.1056/NEJMoa1008433 PMC2976679 20942669

[21] Shen J Ju Z Zhao W . ARID1A deficiency promotes mutability and potentiates therapeutic antitumor immunity unleashed by immune checkpoint blockade. Nat Med. 2018;24:556–562. 10.1038/s41591-018-0012-z PMC6076433 29736026

[22] Jones S Wang TL Shih IM . Frequent mutations of chromatin remodeling gene ARID1A in ovarian clear cell carcinoma. Science (1979). 2010;330:228–231. 10.1126/science.1196333 PMC3076894 20826764

[23] Rumgay H Murphy N Ferrari P . Alcohol and cancer: epidemiology and biological mechanisms. Nutrients. 2021;13:1–13. 10.3390/nu13093173 PMC8470184 34579050

[24] Szabo G Saha B . Alcohol’s effect on host defense. Alcohol Res. 2015;37:159–170. 10.35946/arcr.v37.2.01 PMC4590613 26695755

[25] Xing B Zhang X Gu X . Explore the alterations of downstream molecular pathways caused by ARID1A mutation/knockout in human endometrial cancer cells. J Cancer Res Clin Oncol. 2023;149:17529. 10.1007/s00432-023-05471-x PMC11797191 37906351

